# The first fossil brown lacewing from the Miocene of the Tibetan Plateau (Neuroptera, Hemerobiidae)

**DOI:** 10.3897/zookeys.726.21086

**Published:** 2018-01-10

**Authors:** Qiang Yang, Chaofan Shi, Xiangchuan Li, Hong Pang, Dong Ren

**Affiliations:** 1 State Key Laboratory of Biocontrol, Key Laboratory of Biodiversity Dynamics and Conservation of Guangdong Higher Education Institute, Ecology and Evolution, School of Life Sciences, Sun Yat-sen University, Guangzhou 510275, PR China; 2 College of Life Sciences, Capital Normal University, Xisanhuanbeilu 105, Haidian District, Beijing 100048, PR China; 3 Geoscience Museum, Hebei GEO University, 136 Huaiandonglu, Shijiazhuang, 050031, PR China; 4 School of Earth Science and Engineering, Sun Yat-sen University, Guangzhou 510275, PR China; 5 College of Earth Sciences and Resources & Key Laboratory of Western Mineral Resources and Geological Engineering of the Ministry of Education, Chang’an University, Xi’an 710054, PR China

**Keywords:** Cenozoic, China, *
Wesmaelius
*

## Abstract

A new species of Hemerobiidae, *Wesmaelius makarkini* Yang, Pang & Ren, **sp. n.** is described from the Lower Miocene, Garang Formation of Zeku County, Qinghai Province (northeastern Tibetan Plateau), China. The species is assigned to the widely distributed extant genus *Wesmaelius* Krüger (Hemerobiinae). The species represents the first named fossil of this family from China, which sheds light on the historical distribution of *Wesmaelius* and early divergences within Hemerobiinae.

## Introduction

Hemerobiidae, commonly known as brown lacewings, are the third largest family of Neuroptera, with about 520 species assigned to 27 genera (Oswald 2017). Hemerobiids are the most widely distributed lacewings, from subpolar tundra to tropical regions (Oswald 1993, 2017; Monserrat 2000; Makarkin et al. 2016). The extant brown lacewings have been comprehensively studied by Oswald (1993) including a taxonomic revision, a genus-level phylogeny based on morphology, and the establishment of a subfamilial classification. Recently, Garzón-Orduña et al. (2016) provided a total evidence phylogeny of the family based on combined data of morphological characters and DNA. As a result, seven known subfamilies were recovered to be monophyletic, with the addition of a new subfamily and the revision of Notiobiellinae (Garzón-Orduña et al. 2016).

Compared with its putative sister group Chrysopidae, Hemerobiidae have a relatively sparse and recent fossil record (Haring and Aspöck 2004; Winterton et al. 2010; Wang et al. 2016), although in a recent study, Hemerobiidae are not sister to Chrysopidae, but to the clade including Mantispoidea, Chrysopoidea, and Myrmeleontoidea (Winterton et al. 2017). Only four species have been described from the Mesozoic, with the earliest from the Late Jurassic. All the other 19 species have been described from the Cenozoic, from the Eocene to the Miocene (Makarkin et al. 2003, 2016; Engel and Grimaldi 2007) (Table 1). The Mesozoic hemerobiids comprise four monotypic extinct genera, one from the Late Jurassic of Kazakhstan, two from the Early Cretaceous of Mongolia and England, and one from the Late Cretaceous of Canada (Panfilov 1980; Ponomarenko 1992; Jepson et al. 2012; Klimaszewski and Kevan 1986; Makarkin et al. 2003, 2016). In the Cenozoic, 10 genera have been described from Russia, Baltic amber, Denmark, England, Canada, USA, and Dominican amber (Pictet-Baraban and Hagen 1856; Scudder 1878, 1890; Henriksen 1922; Krüger 1923; Jarzembowski 1980; Makarkin 1991; Oswald 2000; Makarkin et al. 2003; Makarkin and Wedmann 2009; Jepson et al. 2010), as well as China in this paper.

**Table 1.** List of Named Fossil Hemerobiidae (updated from Engel and Grimaldi 2007).

Taxon	Deposit	Reference
*Drepanepteryx oedobia* Makarkin	Miocene, Russia	Makarkin 1991
*Drepanepteryx ramosa* Makarkin	Miocene, Russia	Makarkin 1991
*Hemerobius incertus* Makarkin	Miocene, Russia	Makarkin 1991
*Hemerobius prohumulinus* Makarkin	Miocene, Russia	Makarkin 1991
*Megalomus caucasicus* Makarkin	Miocene, Russia	Makarkin 1991
*Megalomus sikhotensis* Makarkin	Miocene, Russia	Makarkin 1991
*Notiobiella thaumasta* Oswald	Miocene, Dominican amber	Oswald 1999
*Wesmaelius makarkini* sp. n.	Miocene, China	This paper
*Bothromicromus lachlani* Scudder	Oligocene, Canada	Scudder 1878
*Drepanepteryx resinata* (Krüger, 1923)	Eocene, Baltic amber	Krüger 1923; Makarkin et al. 2016
*Megalomus tinctus* (Jarzembowski)	Eocene, England	Jarzembowski 1980; Makarkin 1991
*Prolachlanius resinatus* (Hagen)	Eocene, Baltic amber	Pictet-Baraban and Hagen 1856; Krüger 1923; Makarkin et al. 2012
*Proneuronema gradatum* Makarkin, Wedmann et Weiterschan	Eocene, Baltic amber	Makarkin et al. 2016
*Proneuronema minor* Makarkin, Wedmann et Weiterschan	Eocene, Baltic amber	Makarkin et al. 2016
*Proneuronema wehri* (Makarkin, Archibald et Oswald)	Eocene, USA	Makarkin et al. 2003; Makarkin et al. 2016
*Prospadobius moestus* (Hagen)	Eocene, Baltic amber	Pictet-Baraban and Hagen 1856; Krüger 1923
*Sympherobius completus* Makarkin and Wedmann	Eocene, Baltic amber	Makarkin and Wedmann 2009
*Sympherobius siriae* Jepson, Penney et Green 2010	Eocene, Baltic amber	Jepson et al. 2010
*Wesmaelius mathewesi* Makarkin, Archibald et Oswald 2003	Eocene, Canada	Makarkin et al. 2003
*Megalomus densistriatus* Henriksen	Eocene, Denmark	Henriksen 1922
*Plesiorobius canadensis* (Klimaszewski et Kevan, 1986)	Late Cretaceous, Canadian amber	Klimaszewski and Kevan 1986; Makarkin et al. 2016
*Cretomerobius disjunctus* Ponomarenko	Early Cretaceous, Mongolia	Ponomarenko 1992
*Purbemerobius medialis* Jepson, Makarkin et Coram	Early Cretaceous, England	Jepson et al. 2012
*Promegalomus anomalus* Panfilov	Late Jurassic, Kazakhstan	Panfilov 1980

Fossil hemerobiids have never been described from China. *Mesohemerobius jeholensis* Ping, from the Lower Cretaceous, Yixian Formation of China, was previously placed in Hemerobiidae, but was later excluded from the family by Makarkin et al. (2003) and referred as Neuroptera *incertae sedis*. Wang et al. (2014) mentioned hemerobiids in Fushun amber, but with no descriptions or figures. Herein, we describe a new species of *Wesmaelius* Krüger (Hemerobiidae: Hemerobiinae) from the Lower Miocene of the northeastern Tibetan Plateau in China. The species is the first named fossil of the family Hemerobiidae in China.

## Materials and methods

The specimen was collected from the Guide Group at Caergen Village, Duohemao Town, Zeku County, eastern Qinghai Province, China (34°56'N, 101°48'E, 3700 m a.s.l.) (fig. 1 in Li et al. 2017). The stratum is a papery oil shale deposit and constitutes a lacustrine–fluvial sedimentary succession (fig. 1 in Li et al. 2016), belonging to the Garang Formation (<16–19 Ma), the Lower Miocene. The deposit yielded abundant, exquisitely preserved fossil plants (Guo 1980; Fang et al. 2005, 2007), bird feathers (Yang 1975), and insects including representatives of Hemiptera (Li et al. 2017), Diptera, Hymenoptera, Neuroptera, Mecoptera, Odonata and Coleoptera (pers. obs.). The specimen is housed in the collection of the Key Laboratory of Insect Evolution & Environmental Changes, College of Life Sciences, Capital Normal University, Beijing, China (CNUB; Dong Ren, Curator).

The specimen was examined using a Zeiss Discovery V20 stereomicroscope and photographed with an AxioCam HRc digital camera attached to the Zeiss Discovery V20 stereomicroscope (both instruments Carl Zeiss Light Microscopy, Göttingen, Germany). Line drawings were prepared with the CorelDraw 12 graphics software and with the aid of Adobe Photoshop CS6. The vein terminology in general follows Yang et al. (2012, 2014). Terminology of wing spaces and details of venation (e.g., veinlets, traces, ‘oblique radial branches’ (“ORB”) concept) follows Oswald (1993).

Venation abbreviations used in the text and figures:

**AA1**–**AA3** first to third anterior anal vein;

**CuA** anterior cubitus;

**CuP** posterior cubitus;

**hv** humeral veinlet;

**fl** flexion fold line;

**MA** anterior branche of media;

**MP** posterior branche of media;

**ORB1**, **ORB2**, **ORB3** first to third oblique radial branches;

**RA** anterior radius;

**RP** posterior sector;

**ScA** subcosta anterior;

**ScP** subcosta posterior.

## Systematic palaeontology

### Class Insecta Linnaeus, 1758

#### Order Neuroptera Linnaeus, 1758

##### Family Hemerobiidae Latreille, 1802

###### Subfamily Hemerobiinae Latreille, 1802

####### Genus *Wesmaelius* Krüger, 1922

######## Wesmaelius makarkini

Taxon classificationAnimaliaNeuropteraHemerobiidae

Yang, Pang & Ren sp. n.

http://zoobank.org/4B084F07-9F9E-4EDF-B900-31B2133F1F2F

Fig. 1

######### Holotype.

**Figure 1.**
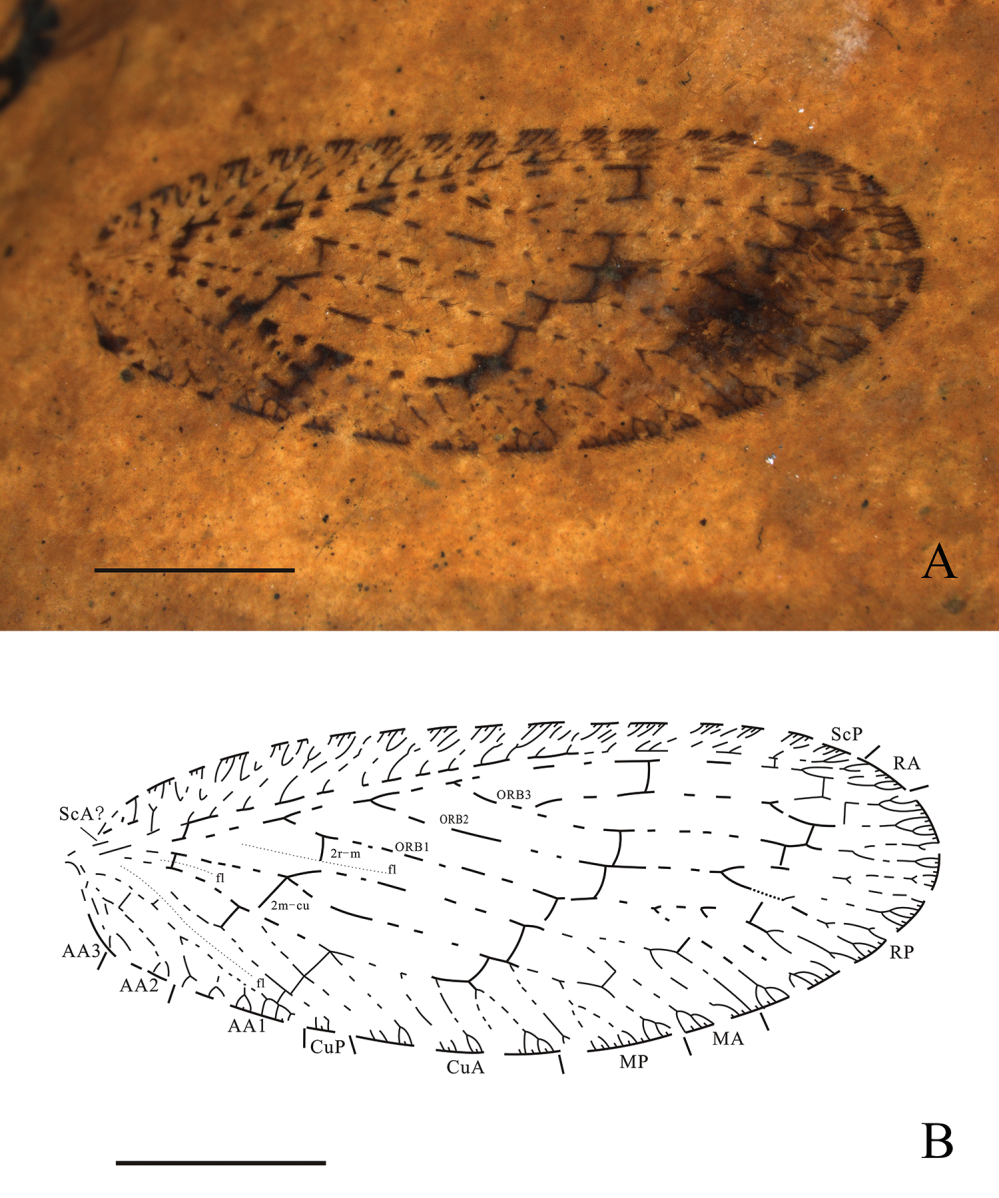
*Wesmaelius makarkini* sp. n., holotype CNU-NEU-QZ2017001. **A** photograph of forewing under alcohol **B** Line drawing of forewing. Scale bars: 2 mm.

CNU-NEU-QZ2017001 (holotype), a complete forewing (Fig. 1).

######### Diagnosis.

Forewing with transparent spots on veins, and dark spots on the graduate crossveins, darker pigmentation along wing margin, subcostal veinlets, and longitudinal veins with dark intervals or dots. MA and MP pectinately forked, 2m-cu located at the fork of MA and M, the crossveins of the third gradate series more oblique.

######### Description.

Holotype CNU-NEU-QZ2017001. Only forewing preserved.

Forewing oval, 8.31 mm long, 3.17 mm wide. Trichosors prominent, along the entire wing margin. Setae distinct, scarce on the veins and dense on the margin. Costal space relatively broad, dilated basally. Humeral veinlet recurrent, with two pectinate branches. Presumable ScA present. Majority of subcostal veinlets branched once, several basal veinlets branched twice, with no crossveins between them. Subcostal space moderately broad, with two prestigmal sc-r crossveins: basal 1sc-r and distal 3sc-r. Posterior trace of RA forked apically, with two distal branches. One RA branch forked once, the other twice. RP with three branches (ORBs) originated from RA; ORB1with two pectinate branches between 3r-m and 4r-m, all with distal forks; ORB2 dichotomously forked between the third and fourth gradate series of crossveins, each branch dichotomously forked; ORB3 forked between the second and third gradate series, with two dichotomously forked branches. M appear to be fused basally with R. M forked at 2m-cu; MA, MP configuration similar, parallel for a long distance, then each with two pectinate branches between the third and fourth gradate series. The second branch of MP dichotomously forked. Forewing with three m-cu crossveins. Crossvein 2r-m present and positioned distally to crossvein 2m-cu; 2m-cu at the fork of MA and MP. Cu divided into CuA and CuP proximal of the first gradate series, close to wing base; CuA with four pectinate branches distal to 2cua-cup, all branches with marginal forks; CuP simple, only with marginal fork. AA1 with three pectinate branches, all with marginal forks. AA2 with two simple branches, forked proximal to aa1-aa2. AA3 simple. Three flexion fold (line) distinct between RP and MA, MP and CuA, CuP and AA1. The third gradate series with nine crossveins and the fourth gradate with seven crossveins. Forewing with transparent spots on veins, and dark spots at the graduate crossveins; margined with darker pigmentation, and no other distinct maculation; wing margin, subcostal veinlets and longitudinal veins with dark intervals or dots.

######### Etymology.

The specific epithet is in honor of the entomologist Dr. Vladimir Nikolaevich Makarkin to acknowledge his great help to the first author in his study of Neuropteran.

######### Type locality and horizon.

Caergen Village, Zeku County, Qinghai Province, China; Garang Formation; The early Miocene.

######### Remarks.

The species can be easily attributed to the genus *Wesmaelius* due to the following characters: two prestigmal sc-r crossveins, three RP branches (ORBs), crossvein 2r-m present and positioned distally to crossvein 2m-cu; intersection of crossvein 2m-cu with M not more than the crossvein’s length distal to fork MA/MP (sometimes anterior to this fork), resulting in cell c2m-cu broad distally; forewing with three m-cu crossveins (Oswald, 1993).

In the genus, *Wesmaelius makarkini* sp. n. is most similar to the extant species of *W. nervosus* (Fabricius, 1793), *W. subnebulosus* (Stephens, 1836) and *W. reisseri* U. Aspöck & H. Aspöck, 1982. The new species with two ORB3 branches, 2m-cu located at the division of MA and MP; while *W. nervosus* with three ORB3 branches, 2m-cu distal to the division of MA and MP; and *W. makarkini* with a distinct large darker pigmentation at the apex of forewing. The new species differs from *W. subnebulosus* and *W. reisseri* in the pectinately forked MA and RP1, 2m-cua located at the division of MA and MP, instead of dichotomously forked MA and RP1, and 2m-cu distal to the division of MA and MP in *W. subnebulosus* and *W. reisseri*. Moreover, the new species has seven crossveins in the fourth series, while *W. reisseri* has four crossveins.

## Discussion

Named fossil Hemerobiidae have been described from the Late Jurassic to the Miocene, including four extinct genera from the Mesozoic and 11 genera from the Cenozoic (Table 1). Among the Cenozoic genera, six of them are extant genera, belonging to five subfamilies (Drepanepteryginae, Sympherobiinae, Megalominae, Hemerobiinae, Notiobiellinae), which are distributed in the three main clades of Hemerobiidae according to Garzón-Orduña et al. (2016). The earliest fossil record of each of the five subfamilies are from the Eocene of Europe and North America, indicating these extant subfamilies have been well differentiated and widely distributed across the Northern hemisphere by the Eocene.

*Wesmaelius* is an extant genus with approximately 62 extant species and two fossil species from the Eocene of Canada (Makarkin et al. 2003) and the Miocene of China (as afore-described). The extant species are widely distributed in the Palearctic, Nearctic, Afrotropic, and Indomalaya, with the majority of species widely distributed across the northern hemisphere; only four species are found in the southern hemisphere (Makarkin et al. 2003; Oswald 2017). Nearly all of them are restricted from the tropical to the temperate zone, and most of them restricted to higher elevation montane region. The genus apparently is distributed from the north to the south, but the center of origin of *Wesmaelius* is questionable, mainly because the generic assignment of the oldest species (i.e., *W. mathewesi*) from the Eocene of Canada is uncertain (Makarkin et al. 2003). Nevertheless, *W. mathewesi* shows high affinity to the genera of *Wesmaelius* and *Hemerobius*, both of which belong to the subfamily Hemerobiinae. Therefore, it represents one of the earliest fossil records of the subfamily to date. *Hemerobius* also has fossil records extending back to the Miocene (Makarkin 1991). The geological history of the Hemerobiinae is still uncertain, due to the undetermined subfamilial assignment of the extinct genera, which requires further study.

## Supplementary Material

XML Treatment for Wesmaelius makarkini
